# Supplementary material for: Siwi cooperates with Par-1 kinase to resolve the autoinhibitory effect of Papi for Siwi-piRISC biogenesis
Source: Nat Commun. 2022 Mar 21;13:1518. doi: 10.1038/s41467-022-29193-9 (PMC8938449; doi:10.1038/s41467-022-29193-9)
Supplement: Supplementary file 1 — Supplementary Information [file 41467_2022_29193_MOESM1_ESM.pdf]

Supplementary Information

Siwi cooperates with Par-1 kinase to resolve the autoinhibitory effect of Papi for Siwi-piRISC biogenesis

Yamada et al.

Table of contents

Supplementary Figures

Supplementary Figure.1 related Figure.1 Phosphorylation of Ser547 of Papi is necessary for Siwi-piRISC biogenesis.

Supplementary Figure 2, related Figure 2. Par-1 kinase is responsible for Papi Ser547 phosphorylation.

Supplementary Figure 3, related Figure 3. Siwi targets cytoplasmic Par-1 kinase to mitochondrial Papi for its phosphorylation.

Supplementary Figure 4, related Figure 4. Auxiliary domain of Papi influences the RNA-binding activity of Papi via the KH domains.

Supplementary table

Supplementary table.1 Sequences of oligonucleotides

Supplementary Fig.1

Yamada et al.

a

Ser157_1st analysis

Sequence: VKVESPK, S5-Phospho (79.96633 Da)
Charge: +2
Monoisotopic m/z: 433.72305 Da, MH+: 866.43883 Da

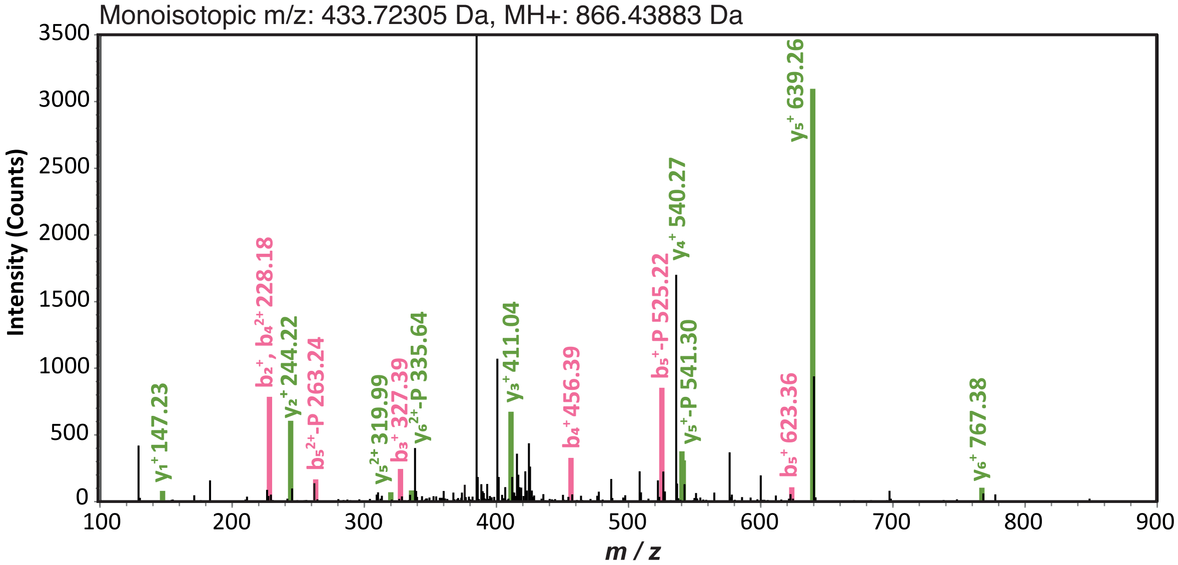

Ser547_1st analysis

Sequence: SDRHPLSISNFDLSYPDPSR, S9-Phospho (79.96633 Da)
Charge: +3
Monoisotopic m/z: 795.02942 Da, MH+: 2383.07370 Da

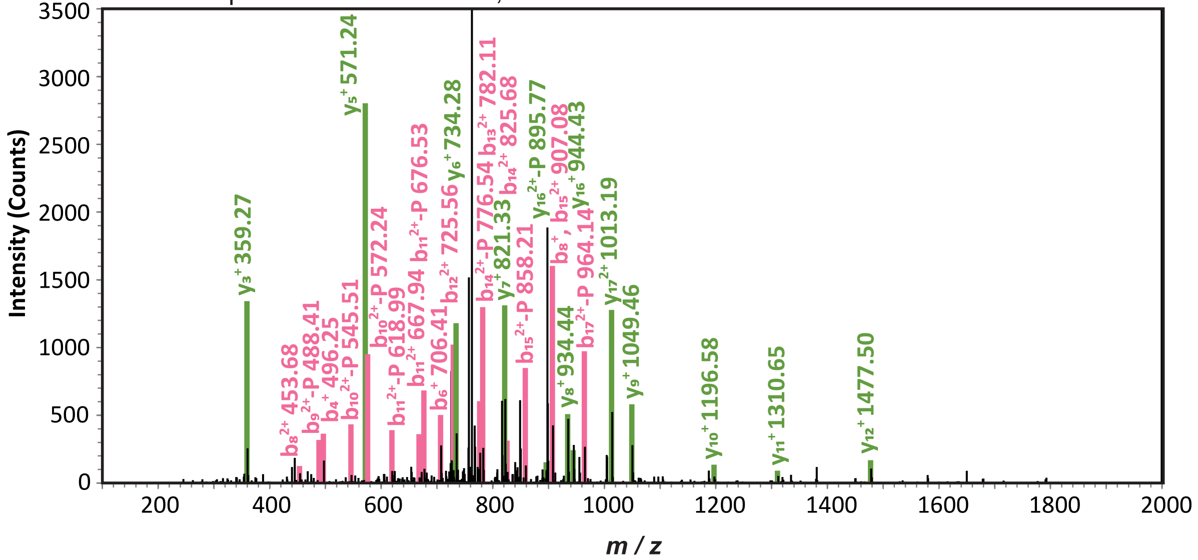

Ser565_1st analysis

Sequence: NKQLNGSDDFLHGER, S7-Phospho (79.96633 Da)
Charge: +3
Monoisotopic m/z: 603.93567 Da, MH+: 1809.79245 Da

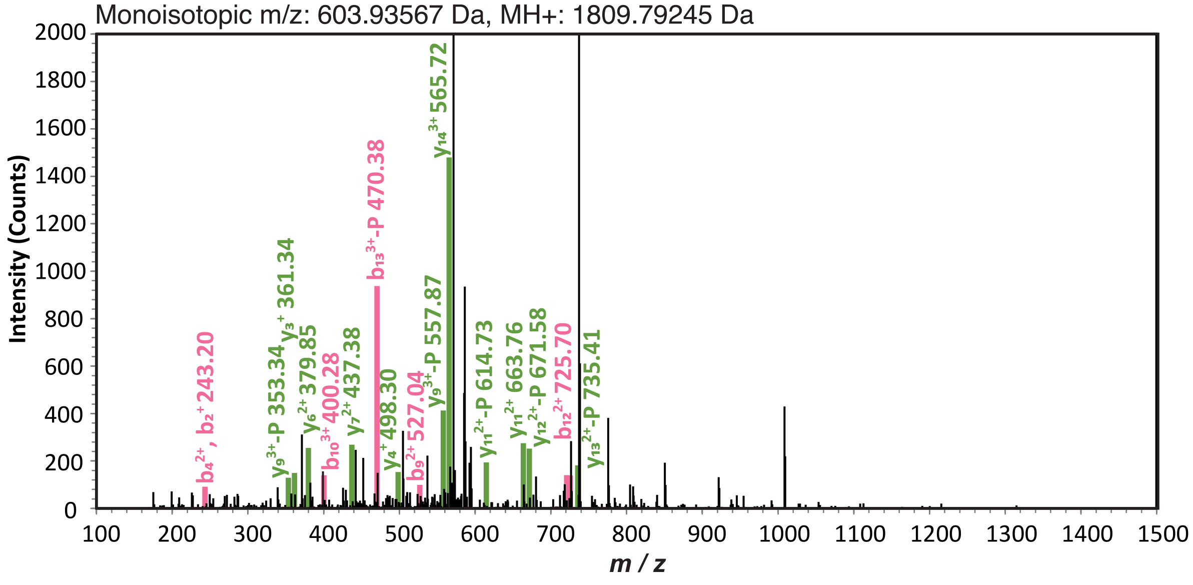

Supplementary Fig.1 Yamada et al.

b

Ser157_2nd analysis

Sequence: VKVESPK, S5-Phospho (79.96633 Da)

Charge: +2

Monoisotopic m/z: 433.72250 Da, MH+: 866.43773 Da

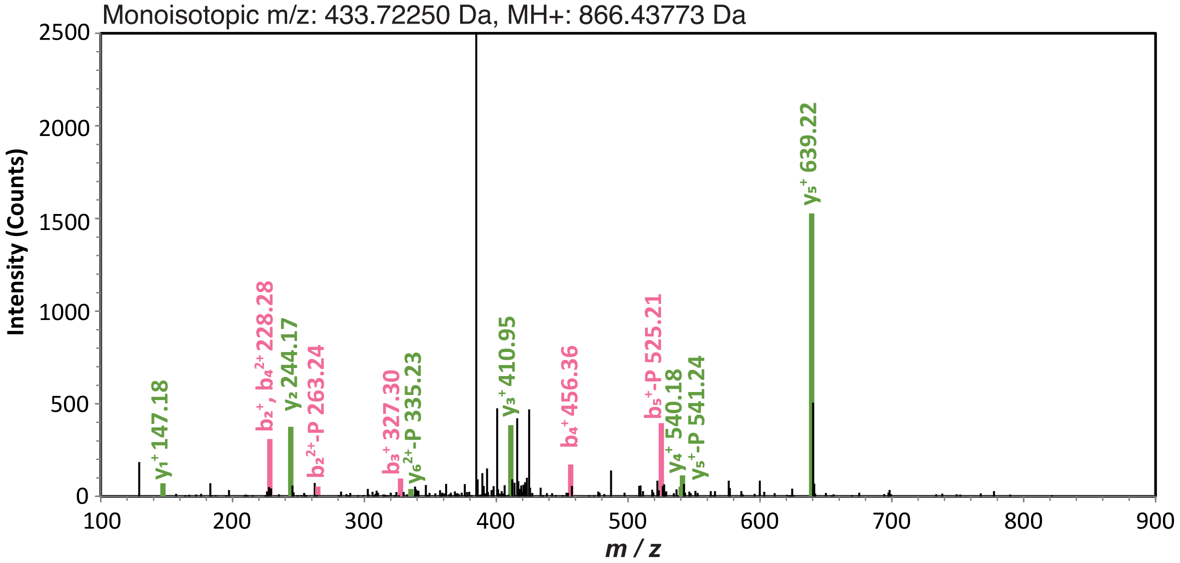

Ser547_2nd analysis

Sequence: SDRHPLSSNFDLSYPDPSR, S9-Phospho (79.96633 Da)

Charge: +3

Monoisotopic m/z: 795.03015 Da, MH+: 2383.07590 Da

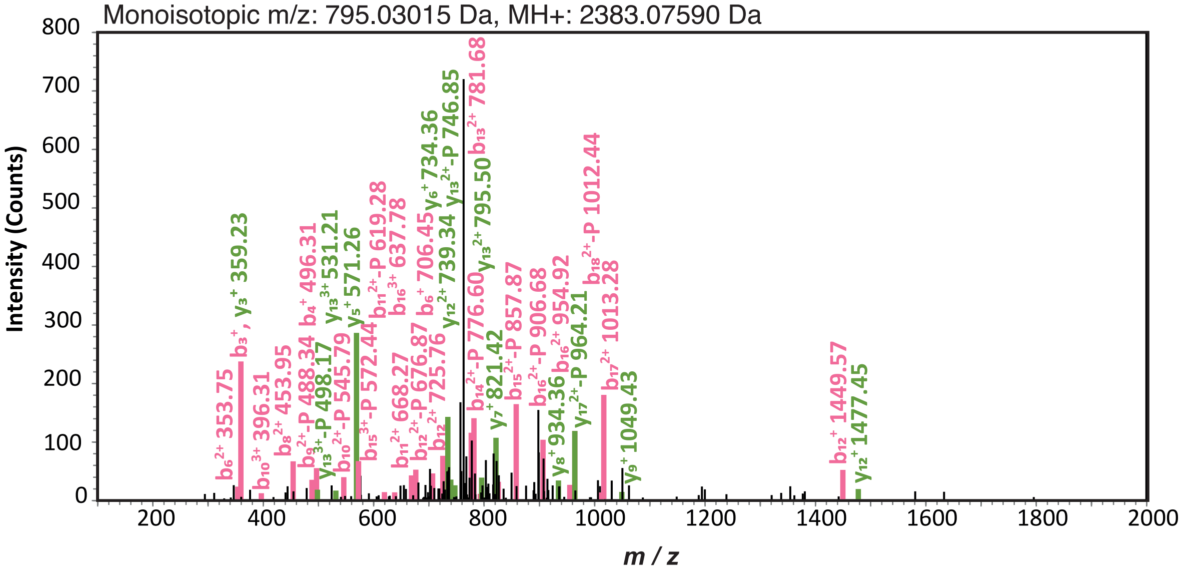

Supplementary Fig.1

Yamada et al.

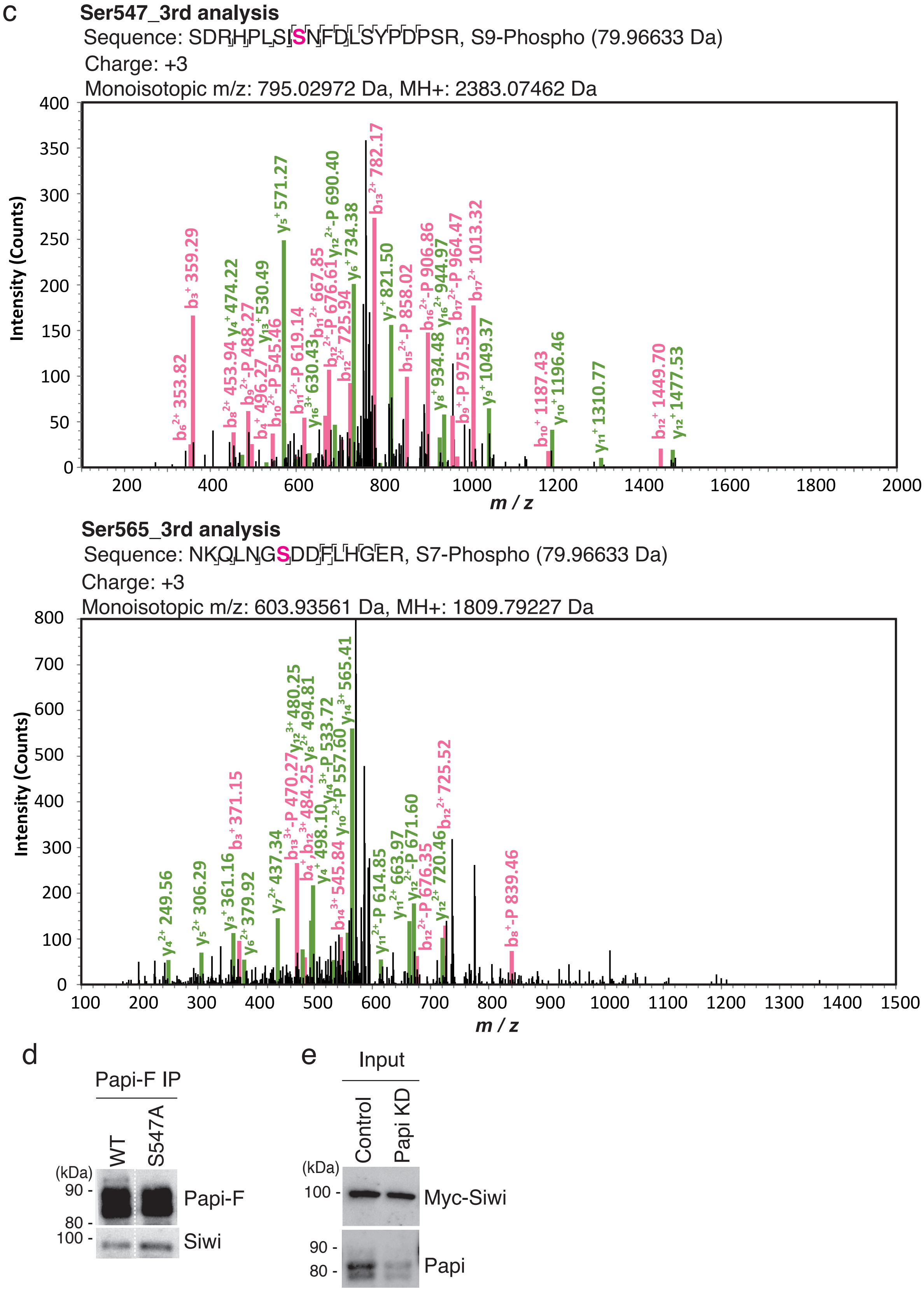

Supplementary Figure 1, related Figure 1. Phosphorylation of Ser547 of Papi is necessary for Siwi-piRISC biogenesis.

(a-c) Phosphopeptide mass spectra showing that Ser157, Ser547, and Ser565 of Papi (indicated with a triangle in Fig. 1b) were phosphorylated. The mass spectra of phosphorylated peptides were recorded on (M + H)$^{+1}$ ions at *m/z* 433.7 for a 7 residue phosphopeptide, (M +3H)$^{+3}$ ions at *m/z* 795.0 for a 20 residue phosphopeptide, and (M + 3H)$^{+3}$ ions at *m/z* 603.9 for a 15 residue phosphopeptide. Red and green in the fragment ion's mass spectra indicate the *b* and *y* ions, respectively. In the first analysis **(a)**, we obtained three phosphopeptides that contain Ser157; VKVES$_{157(P)}$PK (charge = +2, the probability by ptmRS = 100; the confidence was high), Ser547; SDRHPLSIS$_{547(P)}$NFDLSYPDP (+3, 99.96; high), and Ser565; NKQLNGS$_{565(P)}$DDFLHGER (+3, 100; high). The most abundant peak in the spectra, whose *m/z* indicates 384.89, 762.72, and 571.46, respectively, were not assigned, because these represent the unfragmented peptide ions with neutral loss of phosphate. In the second analysis **(b)**, we obtained two phosphopeptides that contain Ser157; VKVES$_{157(P)}$PK (+2, 100; medium) and Ser547; SDRHPLSIS$_{547(P)}$NFDLSYPDP (+3, 99.83; high). The most abundant peak in the spectra which represents neutral loss of phosphate are 384.84 and 762.70. In the third analysis **(c)**, we obtained two phosphopeptides that contain Ser547; SDRHPLSIS$_{547(P)}$NFDLSYPDP (+3, 99.83; high) and Ser565; NKQLNGS$_{565(P)}$DDFLHGER (+3, 100; high). The most abundant peak in the spectra which represents neutral loss of phosphate are 762.52 and 571.43. Information of the PSM, which includes the identified sequence, the site probability of ptmRS, the confidence level, charge, monoisotopic *m/z* and protonated molecule' s mass, are shown above the spectra. The confidence levels 'High' and 'Medium' indicates 1% and 5% FDR, respectively.

(d) Both WT Papi and the S547A mutant bind Siwi in a similar way (*n*=3). Anti-Flag and anti-Siwi antibodies were used for western blotting. Anti-Flag antibody was used for immunoprecipitation of Papi-Flag (Papi-F).

(e) Western blotting shows the abundance of Myc-Siwi and endogenous Papi in Input in Fig. 1e (*n*=3). Source data are provided as a Source Data file.

Supplementary Fig.2

Yamada et al.

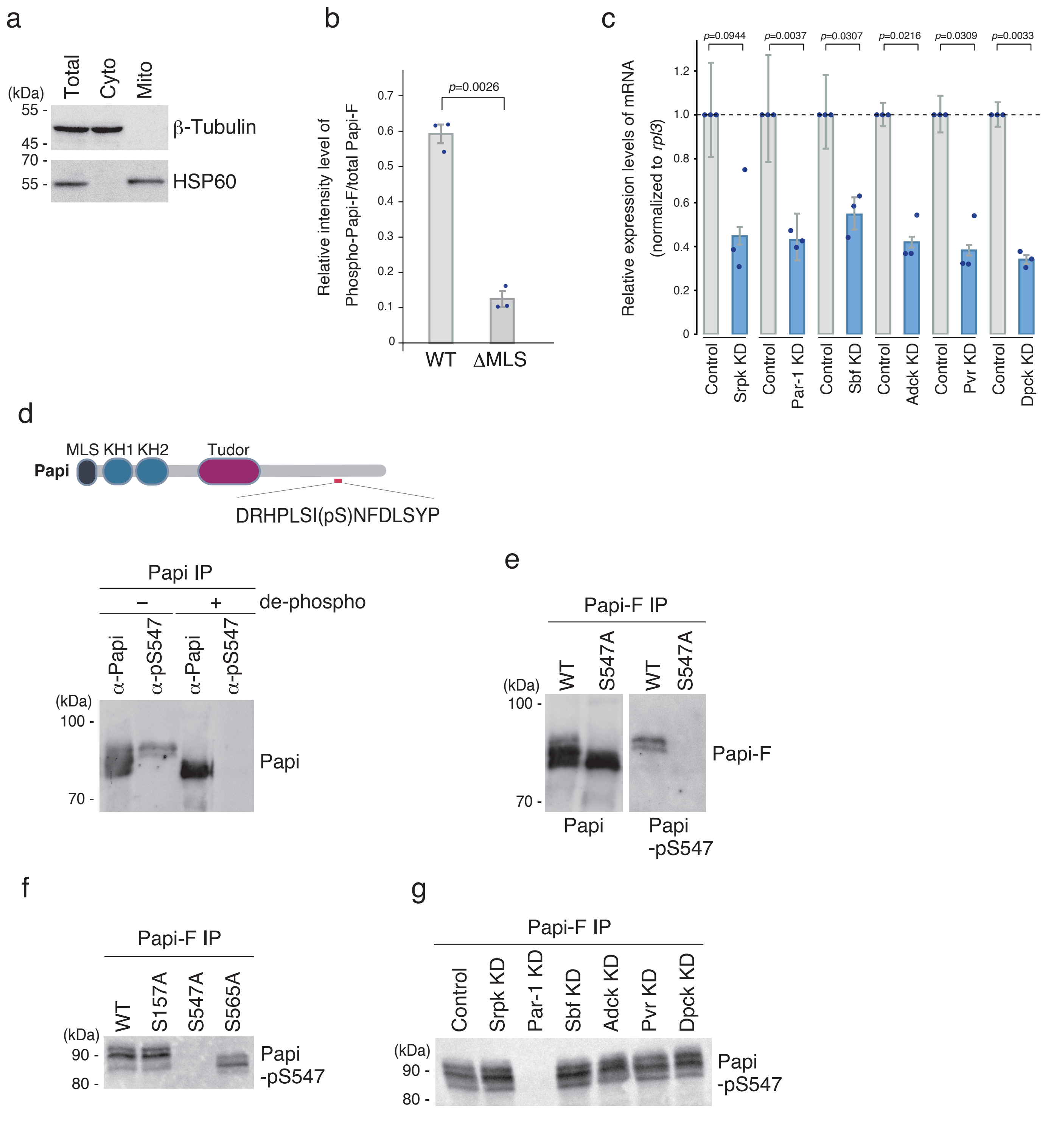

Supplementary Figure 2, related Figure 2. Par-1 kinase is responsible for Papi Ser547 phosphorylation.
(a) Western blotting showing the presence of β-Tubulin in the cytoplasmic (Cyto) fraction and HSP60 in the mitochondrial (Mito) fraction in Fig. 2a (*n*=3).
(b) Bar graph shows the statistical western blotting signals (phospho-Papi-F/total Papi-F) in Fig. 2b (*n*=3). The signal intensities were calculated using ImageJ. Each dot represents the intensity calculated from three independent experiments. *P*-values were calculated by *t*-test (two-sided). Data are presented as mean values +/- SD.
(c) RT-qPCR shows the efficiency of RNAi for the six kinases shown in Fig. 2e (*n*=3). *P*-values were calculated by *t*-test (two-sided). Each dot represents the intensity calculated from three independent experiments. Data are presented as mean values +/- SEM.
(d) Papi immunoisolated from BmN4 cells was dephosphorylated first and then probed with anti-Papi and anti-Papi-pSer547 antibodies (*n*=3). Schematic diagram of Papi showing the epitope used for the production of the anti-Papi-pSer547 antibody.
(e) WT Papi but not the S547A mutant was detected with anti-Papi-pSer547 antibody (*n*=3). This proved the specificity of the antibody for Papi-pSer547.
(f) WT Papi and its mutants shown in Fig. 1d were probed with anti-Papi-pSer547 antibody (*n*=3).
(g) Papi-Flag (Papi-F) immunoisolated from the cellular conditions (Fig. 2f) was probed with anti-Papi-pSer547 antibody (*n*=3).
Source data are provided as a Source Data file.

Supplementary Fig.3

Yamada et al.

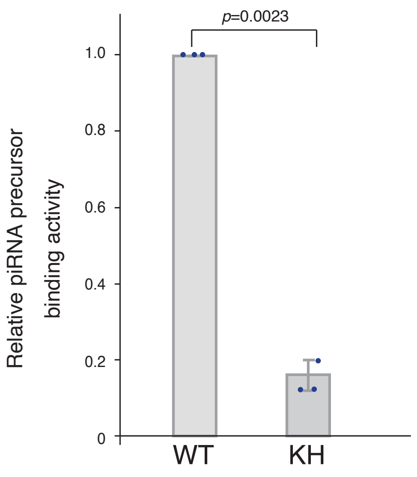

Supplementary Figure 3, related Figure 3. Siwi targets cytoplasmic Par-1 kinase to mitochondrial Papi for its phosphorylation.
Bar graph shows the statistical piRNA precursor signals in Fig. 3i (*n*=3). The signal intensities were calculated using ImageJ. Each dot represents the intensity calculated from three independent experiments. *P*-values were calculated by *t*-test (two-sided). Data are presented as mean values +/- SD.
Source data are provided as a Source Data file.

Supplementary Fig.4 **Yamada et al.**

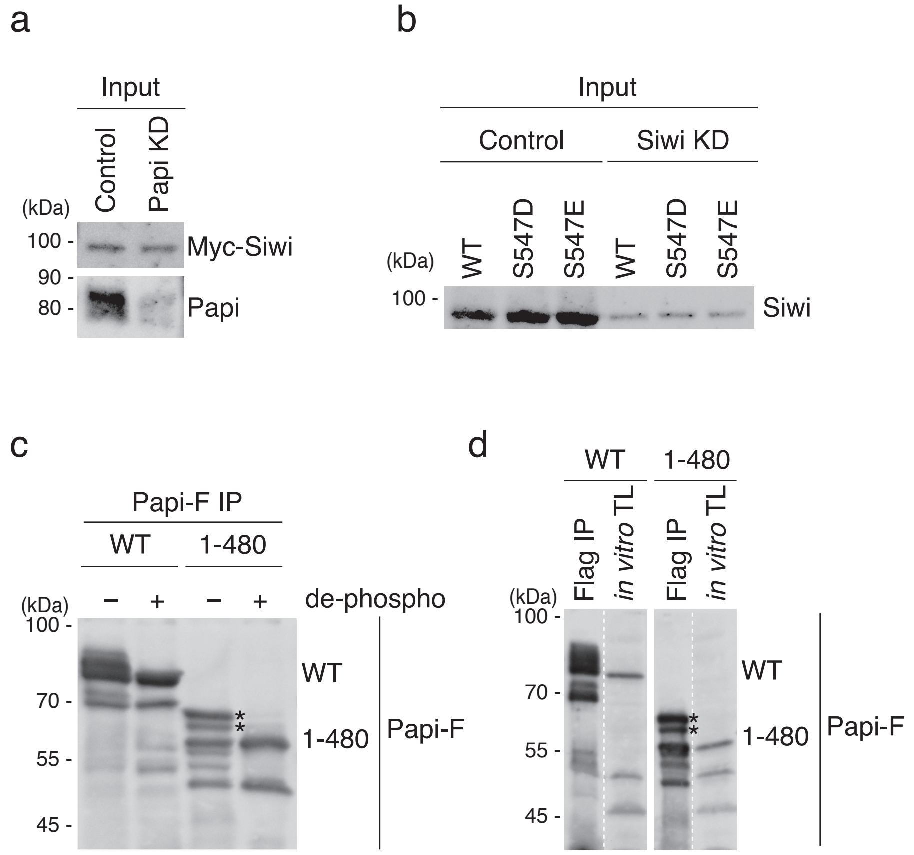

Supplementary Figure 4, related Figure 4. Auxiliary domain of Papi influences the RNA-binding activity of Papi via the KH domains.
(a) Western blotting shows the abundance of Myc-Siwi and endogenous Papi in Input in Fig. 4a (*n*=3).
(b) Western blotting shows the efficiency of Siwi KD in Fig. 4b (*n*=3).
(c) Western blotting shows the Papi1−480 mutant is sensitive to the phosphatase treatment, similar to WT Papi (*n*=3). Asterisks, Phosphorylated Papi1−480.
(d) Western blotting shows the Papi1−480 mutant translated in vitro (in vitro TL: no phosphorylation occurs in the reaction mixtures) migrated with the top band of Papi (+ de-phosphorylation) in (c) (*n*=3). This shows that the two upper bands of the Papi1−480 mutant (− de-phosphorylation) in (c) correspond to post-translationally modified Papi. Asterisks, Phosphorylated Papi1−480. Source data are provided as a Source Data file.

Supplementary Fig.5

Yamada et al.

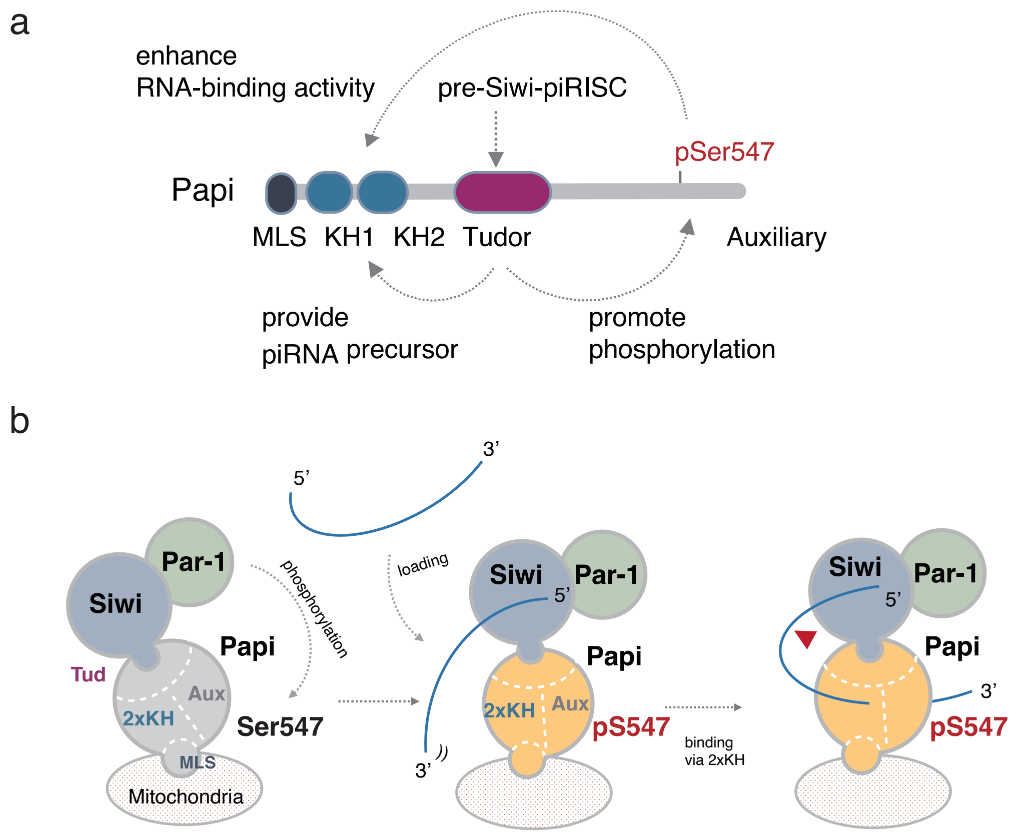

Supplementary Figure 5, related Discussion. Proposed model for Siwi/Par-1-dependent regulation of Papi function and alignment of the Papi and Tdrkh amino acid sequences.

(a) Proposed model for Siwi/Par-1-cooperative regulation of Papi function. MLS, mitochondrial localization signal; KH1 and KH2, K-homology domains.

(b) Par-1-mediated Papi phosphorylation at Ser547 alters the folding and/or other aspects of Papi (indicated by the color change), allowing Papi to bind Siwi-bound piRNA precursors via the KH domains. This molecular scheme is important for Zuc-mediated processing (red triangle).

(c) Multiple sequence alignment of *Bombyx* and *Drosophila* Papi (BmPapi and DmPapi) and mouse Tdrkh. Conserved residues across the three species are boxed in gray. The KH domains are boxed in blue; the Tudor domain is boxed in purple; Ser547 in BmPapi is shown in turquoise blue; the MLS in BmPapi is shown by red letters; N-IDR and C-IDR are highlighted in green. (d) Plots show that BmPapi and Tdrkh both have two IDRs (N-IDR and C-IDR). The IDRs were predicted using DISOPRED3 (http://bioinf.cs.ucl.ac.uk/psipred/). Schematic drawing showing the domain structure of BmPapi and Tdrkh. Source data are provided as a Source Data file.

Supplementary Fig.5

Yamada et al.

c

```
BmPapi  --------MSLNTKLALPIALGLSLVTVTAFVAYYVLKKDEEENDNKTVR----------
DmPapi  --MLRNTPFGATPTYKLLLGFGLCSLGGAMLYAYFKTRNDEEEADSGGQRPASGIRGQTE
Tdrkh   MSTERTSWTNLSTIQKIALGLGIPASATVAYILYRRYRESREERLT--------------

BmPapi  ---ITKINTIEIHVPKSIVPALIGRNGSNIKDLQKKSGAQIHFKKFTDQDYDVCVVRGRA
DmPapi  EQKPQKEVCLKIVVDNEHVPLIMGRGGSNIKLIEEKTLAKIRLRD-KDSGHKFCDISGVP   KH1
Tdrkh   -FVGEDDIEIEMRVPQEAVKLIIGRQGANIKQLRKQTGARIDVDTEDVGDERVLLISGFP

BmPapi  DTTQLAETLIHDFIKQQPTIMSESITVPSWSCGRIIGSGGENVNDISHRSGARVKVESPK
DmPapi  DAVKAARALLIKEIERAPVV-KVELQVPQRLASKINGRGGELLQEIRSSSLAKLNIDLNG   KH2
Tdrkh   VQVCKAKAAIHQILTENTPV-FEQLSVPQRSVGRIIGRGGETIRSICKASGAKITCDKES

BmPapi  STDKVAEHLVTFRGTKEQIEVAKKLVENCISL----ERCRREIEQSKRPPRHSSSPPSP-
DmPapi  RN---GKAKITIIGNQKQVNIARKMLDDQIEEDEELVRSMEEVEQRREPRRSPTNSIAS-
Tdrkh   EGTLLLSRLIKISGTQKEVAAAKHLILEKVSEDEELRKRIAHSAETRVPRKQPISVRREE

BmPapi  ------------------CPSPG------------DRDADADAQGDVTHTRVKY------
DmPapi  ------------------SM-----------------YSSQTSLSSHTQPRDKL------   N-IDR
Tdrkh   VTEPGGAGEAALWKNTNSSMGPATPLEVPLRKGGGDMVVVGPKEGSWEKPNDDSFQNSGA

BmPapi  --------------KRPETTGPSIEVYVSAVSSPSRFWVQFVGPQVAQLDDLVAHMTEYY
DmPapi  --------------MASKGEGKPMEVYVSAVASPTKFWVQLIGPQSKKLDSMVQEMTSYY
Tdrkh   QSSPETSMFEIPSPDFSFHADEYLEVYVSASEHPNHFWIQIIGSRSLQLDKLVSEMTQHY

BmPapi  SKKENREAHTLRHVSVGQVVAAVFRHDGRWYRARVHDIRPNEFDSSQQVADVFYLDYGDS
DmPapi  SSAENRAKHVLTAPYVGQIVAAVFKFDEKWYRAEIVDIMPNQYNPKEQVIDLYFVDYGDS   Tudor
Tdrkh   ENSLPED----LTVHVGDIVAAPLSTNGEWYRARVLGTLEN------GNLDLYFVDFGDN

BmPapi  EYVATHELCELRADLLRLRFQAMECFLAGVRPASGEEAVSPSGQRWDKWHPQAVERFEEL
DmPapi  EYISPADICELRTDFLTLRFQAVECFLANVKSTIQTEP--------ITWPKSSIAKFEEL
Tdrkh   GDCALKDLRALRSDFLSLPFQAIECSLARIAPTG------------EEWEEEALDEFDRL

BmPapi  TQVARWKALVSRTCTYKKTATA------EGEKDKEIPGIKLFDVTDEGELDVGAVLVAEG
DmPapi  TEVAHWRKLIARVVTYKERPRATTAVSAAAKEGTPLPGVELFDPADNSELNIADLMITQG
Tdrkh   THCADWKPLVAKISSYVQTGIS------------TWPKIYLYDTSDEKKLDIGLELVRKG

BmPapi  WAVAGPAPSPRPSP----PRGHTTPFGDLSKSKVL-SMTGGGGRSSSVPKDHKDDGSNVL
DmPapi  FALPLDDSYPVRSRSSTPSSNSDSTIEELCVSNPVTPLTPHSPMSMSIDVDSIT------   C-IDR
Tdrkh   YAVELPEDMEENRTVPNMLKDMATETDD-SLASIL-TETKKSPEEMPHTLSCLSL-SEAA

BmPapi  TVEGDDSKDKDGITASKSLASGLEKSDRHPLSISNFDLSYPDPSRNKQLNGSDDFLHGER   S547
DmPapi  QAENEHL--AQQLQHLQHKLNGNDIKNINPAKLTATDLENGNNNNASTTNGASAH-----
Tdrkh   SMSGDDNLEDDLF-----------------------------------------------

BmPapi  QNIDNEITIDTLVPPSPLSNAPILSKTLQDEFKANMNRIDSHHSNLENLVKSAFEK  629
DmPapi  --------------------------------------------------------  576
Tdrkh   --------------------------------------------------------  560
```

d

BmPapi

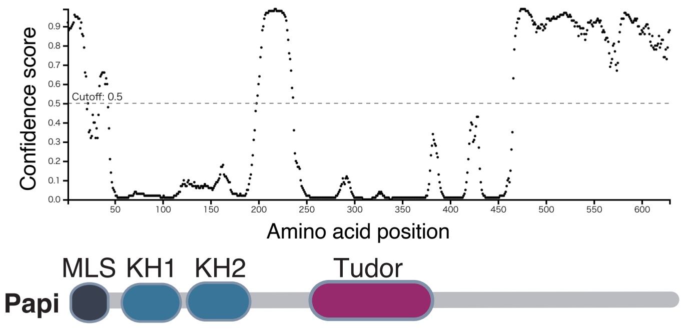

Tdrkh

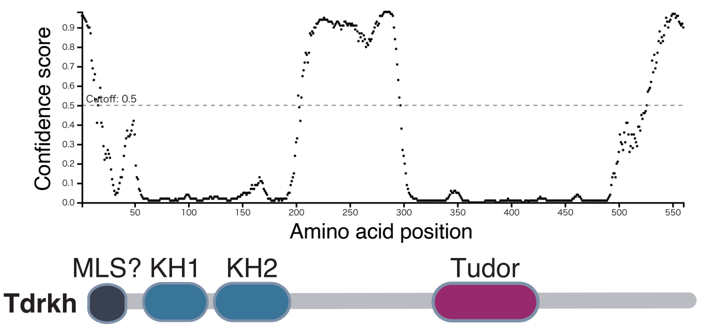

Supplementary Table 1. Sequences of oligonucleotides

Experiment	Name	Sequence*
Plasmid Construction	Papi-Flag-S157A-F	GAGGCACCCAAGTCAACAGATAAAG
	Papi-Flag-S157A-R	CACTTTGACGCGAGCGCCACTGCGA
	Papi-Flag-S547A-F	GCGAACTTCGATCTATCGTATCCCG
	Papi-Flag-S547A-R**	TATCGATAACGGGTGGCGGTCAGAC
	Papi-Flag-S565A-F	GCCGATGATTTCTTGCACGGCGAGC
	Papi-Flag-S565A-R	GCCGTTCAGTTGTTTGTTCCTCGAA
	Papi-Flag-ΔMLS-F	ATGAAAATAAATACAATTGAAATTCATGTTCCTAAAAGCA
	Papi-Flag-ΔMLS-R	AAGCTTTAAATTCGAACCGCGGGCC
	Papi-Flag-S547D-F	GACAACTTCGATCTATCGTATCCCG
	Papi-Flag-S547D-R**	TATCGATAACGGGTGGCGGTCAGAC
	Papi-Flag-S547E-F	GAGAACTTCGATCTATCGTATCCCG
	Papi-Flag-S547E-R**	TATCGATAACGGGTGGCGGTCAGAC
	Papi-Flag-1-222-F***	GAGGGCCCGCGGTTCGAAGACTACA
	Papi-Flag-1-222-R	GTCGCGGTCGCCGGGCGAGGGGCAG
	Papi-Flag-1-480-F***	GAGGGCCCGCGGTTCGAAGACTACA
	Papi-Flag-1-480-R	GAAGGGCGTGGTGTGTCCGCGCGGG
	Par-1-cDNA-F	ATGTACGAAGTTTCCTGCG
	Par-1-cDNA-R	TCACAGCTTGAGTTCGTTCG
	Par-1-HiFi-F	CCCAGCGCTGGATCCATGTACGAAGTTTCCTGCGTCGCCG
	Par-1-HiFi-R	CCGCGGGCCCTCTAGTCACAGCTTGAGTTCGTTCGCTATC
	LacZ-HiFi-F	CCCAGCGCTGGATCCATGATAGATCCCGTCGTTTT
	LacZ-HiFi-R	CCGCGGGCCCTCTAGttaTTCGAATGGGTGACCTC
	3 × Flag-F	CTAGAGGGCCCGCGGTTCGAAGGTA
	3 × Flag-R	GGATCCAGCGCTGGGCTGCAGGAAT
	pET-28a(+)-F	TGAGATCCGGCTGCTAACAAAGCCC
	pET-28a(+)-R	GGTATATCTCCTTCTTAAAGTTAAA
	HiFi-pET-28a(+)-BmPapi-3xFlag-F	AGAAGGAGATATACCATGTCATTGAACACAAAATT
	HiFi-pET-28a(+)-BmPapi-3xFlag-R	AGCAGCCGGATCtcaGATATCCTTGTCATCGTCAT
	HiFi-pET-28a(+)-BmPapi-ΔMLS-3xFlag-F	AGAAGGAGATATACCATGAAAATAAATACAATTGAAATTCATGTTCCTAAAAGCA
	HiFi-pET-28a(+)-BmPapi-ΔMLS-3xFlag-R	GAACCGCGGGCCCTCCTTTTCAAAAGCGGACTTAACGAGG
dsRNA production	T7-dsLuc-F	TAATACGACTCACTATAGGGGGAGAGCAACTGCATAAGGC
	T7-dsLuc-R	TAATACGACTCACTATAGGGTCCCTATCGAAGGACTCTGG
	T7-dsSRPK-F	TAATACGACTCACTATAGGGCACGTCACTCGGAAGCTAGG
	T7-dsSRPK-R	TAATACGACTCACTATAGGGTGAGGCAACCTTAGCCCCAA
	T7-dsPar-1-F	TAATACGACTCACTATAGGGAAGCTCTTCCAAGTTATAGA
	T7-dsPar-1-R	TAATACGACTCACTATAGGGAGATTCTCGCAGTCCGTTGA
	T7-dsSbf-F	TAATACGACTCACTATAGGGGTTATTGACGAGGAAGAGGA
	T7-dsSbf-R	TAATACGACTCACTATAGGGCATCGCTCGTGACACGCGGC
	T7-dsAdck-F	TAATACGACTCACTATAGGGTCATAAAGCAAAATTAAAAG
	T7-dsAdck-R	TAATACGACTCACTATAGGGAATAAACTGTTACATCCTTG
	T7-dsPvr-F	TAATACGACTCACTATAGGGCTGCCTTTTAACGAATACTT
	T7-dsPvr-R	TAATACGACTCACTATAGGGATGACAGTTCATGCTCCTCG
	T7-dsDpck-F	TAATACGACTCACTATAGGGTTGCTCGAAAAGTTTTAGAA
	T7-dsDpck-R	TAATACGACTCACTATAGGGCTTTGATTTTCTTAAGACTT
qRT-PCR	rpl3-F	GGTGTCACCAAGGGCAAAGG
	rpl3-R	AGGATGCCAAGCTCCAATGC
	Srpk-F	AATAGAAGAGTACTCGCCATACAGG
	Srpk-R	TCTAGACGATTCTGGTTCTGGTTAC
	Par-1-F	CTAGAGAGCATAATGCGAGACAAAT
	Par-1-R	CAGGGAGTATTCAATCTCTTGTCTG
	Sbf-F	GCGAACAGAATGGTTTACTTGTTAC
	Sbf-R	GACACTGTTGGTGATACTATTGCTG
	Adck-F	CCTCAAAACACAGTCGAAGAACTAT
	Adck-R	TGCTTTATGAACTTGTGCTAGTGAC
	Pvr-F	CAATGACTCAGTGGAAGTATCTGTG
	Pvr-R	ATACTCCATTGTGTTTCTTGGTAGC
	Dpck-F	ATGTTCATTGTTGGACTGACTGGT
	Dpck-R	CTTCATCGGCATCTATTACGG
RNAi	siLuc sense	*CGUACGCGGAAUACUUCGA*TT
	siLuc antisense	*UCGAAGUAUUCCGCGUACG*TT
	siPapi sense	*GGUCGAAAGUCCUAAAAGU*TT
	siPapi antisense	*ACUUUUAGGACUUUCGACC*TT
	siSiwi sense	*CACUCGGAGGAUAUCUCUU*TT
	siSiwi antisense	*AAGAGAUAUCCUCCGAGUG*TT
	siPar-1 sense	*GAAAUUCCUCGUCCUGAAU*TT
	siPar-1 antisense	*AUUCAGGACGAGGAAUUUC*TT
Northern blotting	RT3-1 piRNA	ACCAGCCGATCGTC

*: RNA is shown in itallics; **: identical sequences; ***: identical sequences.
