# Supplementary material for: Siwi cooperates with Par-1 kinase to resolve the autoinhibitory effect of Papi for Siwi-piRISC biogenesis
Source: Nat Commun. 2022 Mar 21;13:1518. doi: 10.1038/s41467-022-29193-9 (PMC8938449; doi:10.1038/s41467-022-29193-9)
Supplement: Supplementary file 2 — Reporting Summary [file 41467_2022_29193_MOESM2_ESM.pdf]

nature portfolio

Corresponding author(s): Mikiko C. Siomi

Last updated by author(s): Feb 2, 2022

Reporting Summary

Nature Portfolio wishes to improve the reproducibility of the work that we publish. This form provides structure for consistency and transparency in reporting. For further information on Nature Portfolio policies, see our Editorial Policies and the Editorial Policy Checklist.

Statistics

For all statistical analyses, confirm that the following items are present in the figure legend, table legend, main text, or Methods section.

n/a	Confirmed	
☐	☒	The exact sample size (n) for each experimental group/condition, given as a discrete number and unit of measurement
☐	☒	A statement on whether measurements were taken from distinct samples or whether the same sample was measured repeatedly
☐	☒	The statistical test(s) used AND whether they are one- or two-sided<br>*Only common tests should be described solely by name; describe more complex techniques in the Methods section.*
☒	☐	A description of all covariates tested
☒	☐	A description of any assumptions or corrections, such as tests of normality and adjustment for multiple comparisons
☐	☒	A full description of the statistical parameters including central tendency (e.g. means) or other basic estimates (e.g. regression coefficient) AND variation (e.g. standard deviation) or associated estimates of uncertainty (e.g. confidence intervals)
☐	☒	For null hypothesis testing, the test statistic (e.g. F, t, r) with confidence intervals, effect sizes, degrees of freedom and P value noted<br>*Give P values as exact values whenever suitable.*
☒	☐	For Bayesian analysis, information on the choice of priors and Markov chain Monte Carlo settings
☒	☐	For hierarchical and complex designs, identification of the appropriate level for tests and full reporting of outcomes
☒	☐	Estimates of effect sizes (e.g. Cohen's d, Pearson's r), indicating how they were calculated

Our web collection on statistics for biologists contains articles on many of the points above.

Software and code

Policy information about availability of computer code

Data collection	Proteome Discoverer 2.1 (Thermo Fisher Scientific), StepOne software v2.2.2 (Thermo Fisher Scientific), ChemiDoc XRS Plus System (Bio-Rad), ImageJ (National Institutes of Health, version 1.53a), R (version 4.0.5)
Data analysis	For analyzing the reads obtained from deep sequencing analysis, cutadapt (version 1.14), Bowtie (version 1.1.1), fastx-collapser from FASTX-Toolkit (version 0.0.14), and STAR (version 2.5.3a) were used.

For manuscripts utilizing custom algorithms or software that are central to the research but not yet described in published literature, software must be made available to editors and reviewers. We strongly encourage code deposition in a community repository (e.g. GitHub). See the Nature Portfolio guidelines for submitting code & software for further information.

Data

Policy information about availability of data

All manuscripts must include a data availability statement. This statement should provide the following information, where applicable:

- Accession codes, unique identifiers, or web links for publicly available datasets
- A description of any restrictions on data availability
- For clinical datasets or third party data, please ensure that the statement adheres to our policy

The FAST-iCLIP data have been deposited in the Gene Expression Omnibus under accession number GSE179432 (https://www.ncbi.nlm.nih.gov/geo/query/acc.cgi?acc=GSE179432)
The mass spectrometry data have been deposited to the proteomeXchange Consortium via the JPOST repository PXD027395 (http://proteomecentral.proteomexchange.org/cgi/GetDataset?ID=PXD027395) (Phosphorylation sites that influences the RNA-binding activity of Papi) and PXD027396 (http://proteomecentral.proteomexchange.org/cgi/GetDataset?ID=PXD027396) (Papi interacting proteins in mitochondrial fraction of BmN4 cells).

Field-specific reporting

Please select the one below that is the best fit for your research. If you are not sure, read the appropriate sections before making your selection.

- [x] Life sciences
- [ ] Behavioural & social sciences
- [ ] Ecological, evolutionary & environmental sciences

For a reference copy of the document with all sections, see nature.com/documents/nr-reporting-summary-flat.pdf

Life sciences study design

All studies must disclose on these points even when the disclosure is negative.

Sample size	For WB, IP, CLIP, FAST-iCLIP-seq and RT-qPCR experiments the sample size was chosen based on data from our previous work.
Data exclusions	No data exclusion in this manuscript.
Replication	Experiments were repeated as described in the figure legends. All experiments shown could be reproduced as described.
Randomization	This study does not involve animal experiments. Cells were cultured in flasks or dishes and when sufficient cultured cells for the each specific assay were available flasks or dishes were randomly chosen for transfection or knockdown.
Blinding	Experiments performed in this article were not blinded. Researchers were not blinded during data collection or analysis. This was not possible since, transfection and knockdown of the cells was conducted by the same reserchers.

Reporting for specific materials, systems and methods

We require information from authors about some types of materials, experimental systems and methods used in many studies. Here, indicate whether each material, system or method listed is relevant to your study. If you are not sure if a list item applies to your research, read the appropriate section before selecting a response.

Materials & experimental systems

n/a	Involved in the study	
[ ]	[x]	Antibodies
[ ]	[x]	Eukaryotic cell lines
[x]	[ ]	Palaeontology and archaeology
[x]	[ ]	Animals and other organisms
[x]	[ ]	Human research participants
[x]	[ ]	Clinical data
[x]	[ ]	Dual use research of concern

Methods

n/a	Involved in the study	
[x]	[ ]	ChIP-seq
[x]	[ ]	Flow cytometry
[x]	[ ]	MRI-based neuroimaging

Antibodies

Antibodies used

[Primary antibodies]
Anti-Papi and Siwi monoclonal antibodies were produced from immunized mice, respectively (Nishida et al., 2018, Nature 555, 260-264; Nishida et al., 2015, Cell Reports 10, 193-203). AntiPapi-pS547 monoclonal antibodies were produced from immunized mice in this paper.
Anti-Flag M2 (catalog number: F3165, Sigma), anti-Flag produced in rabbit (catalog number: F7425, Sigma), anti-Myc (9E10, Developmental Studies Hybridoma Bank), anti-β-Tubulin (E7, Developmental Studies Hybridoma Bank), and anti-HSP60 (LK1, catalog number: SMC-110B, StressMarq Biosciences) monoclonal antibodies were purchased.
For western blotting, each antibody was used at the following dilution [anti-Papi (1:1000), Siwi (1:5000), Papi-S547 (supernatant of hybridoma cells), Flag (1:1000), Myc (1:1000), β-Tubulin (1:1000), HSP60 (1:1000)].
[Secondary antibodies]
Peroxidase-conjugated anti-mouse IgG antibody (1:5,000 dilution; catalog number: 55558, Cappel)
Peroxidase-conjugated anti-rabbit IgG antibody (1:1,000 dilution; catalog number: 7074S, Cell Signaling Technology)

Validation

[Primary antibodies]
Anti-Papi and Siwi monoclonal antibodies were validated by Western blotting of a knockdown lysate of BmN4 in a previous studies, respectively (Nishida et al., 2018, Nature 555, 260-264; Nishida et al., 2015, Cell Reports 10, 193-203). Papi-pS547 monoclonal antibodies was validated by Western blotting of lysates of normal BmN4 and transected BmN4 in this study (Fig. S2d, e).
For the following purchased antibodies, see manufacturer information:
Anti-Flag M2 (https://www.sigmaaldrich.com/certificates/COFA/F3/F3165/F3165-BULK________SLCG2330__.pdf)
Anti-Flag produced in rabbit (https://www.sigmaaldrich.com/certificates/Graphics/COfAInfo/sigmail01/pdf/F7425_064M4757V.pdf)
Anti-Myc (https://dshb.biology.uiowa.edu/9E-10)
Anti-β-Tubulin (https://dshb.biology.uiowa.edu/E7_2)

Anti-HSP60 (https://www.stressmarq.com/products/antibodies/hsp60-antibody-smc-110/?v=3e8d115eb4b3)
[Secondary antibodies]
Peroxidase-conjugated anti-mouse IgG antibody (https://www.mpbio.com/jp/0855558-peroxidase-conjugated-sheep-igg-fraction-to-mouse-igg-no-cross-to-human)
Peroxidase-conjugated anti-rabbit IgG antibody (https://www.cellsignal.jp/products/secondary-antibodies/anti-rabbit-igg-hrp-linked-antibody/7074)

Eukaryotic cell lines

Policy information about cell lines

Cell line source(s)	BmN4 cells were gifted from National Institute of Agrobiological Sciences (NIAS).
Authentication	BmN4 cell have not been authenticated.
Mycoplasma contamination	BmN4 cells were not tested for mycoplasma contamination.
Commonly misidentified lines (See ICLAC register)	BmN4 cells are not misidentified cell lines.
