# Supplementary material for: Siwi cooperates with Par-1 kinase to resolve the autoinhibitory effect of Papi for Siwi-piRISC biogenesis
Source: Nat Commun. 2022 Mar 21;13:1518. doi: 10.1038/s41467-022-29193-9 (PMC8938449; doi:10.1038/s41467-022-29193-9)

Original source data of blotting and silver staing images of Figure.1

Fig. 1a

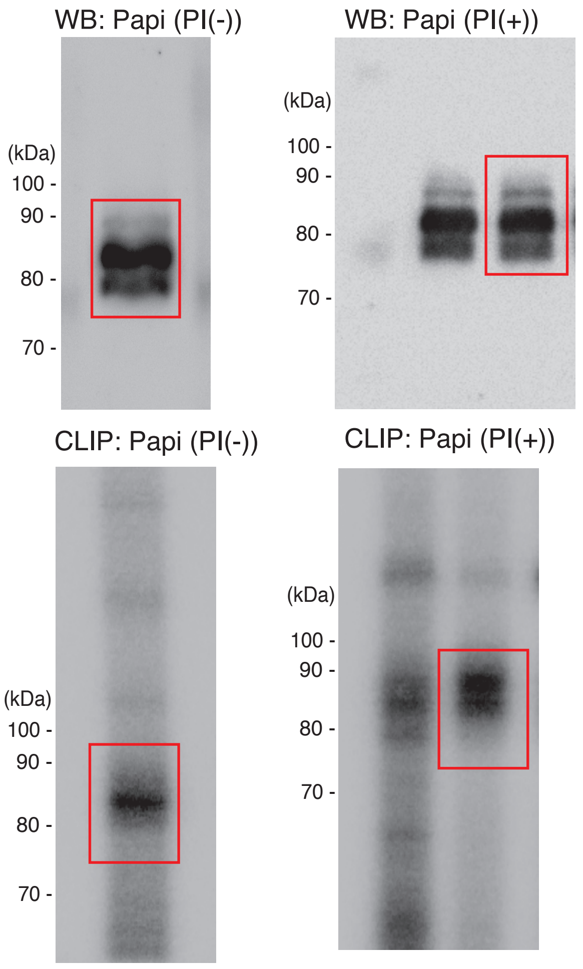

Fig. 1b

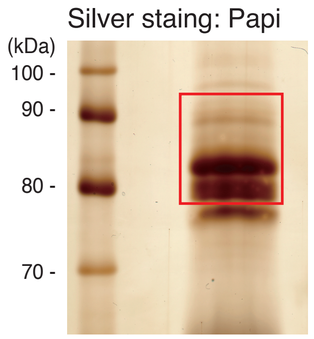

Fig. 1c

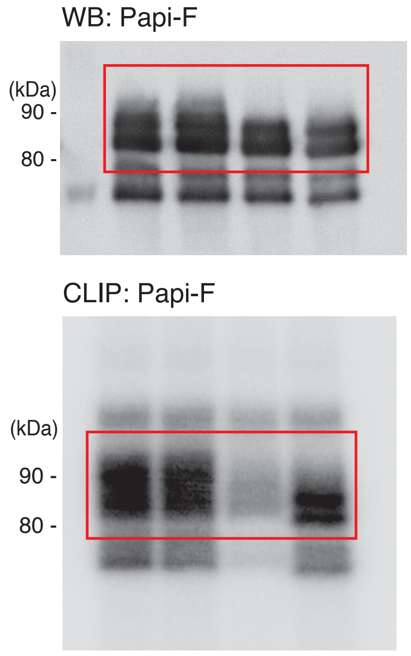

Fig. 1e

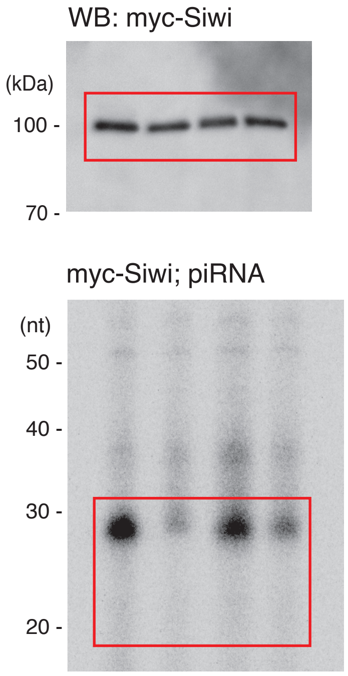

Original source data of blotting and silver staing images of Figure.2

Fig. 2a

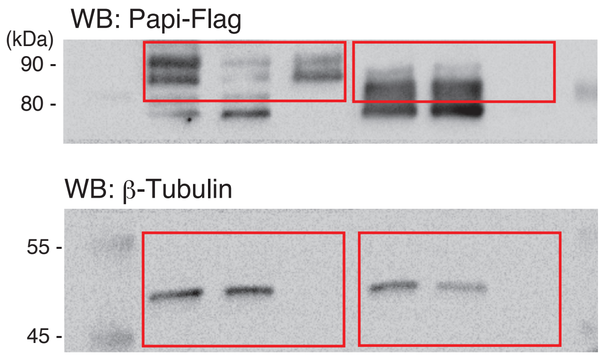

Fig. 2b

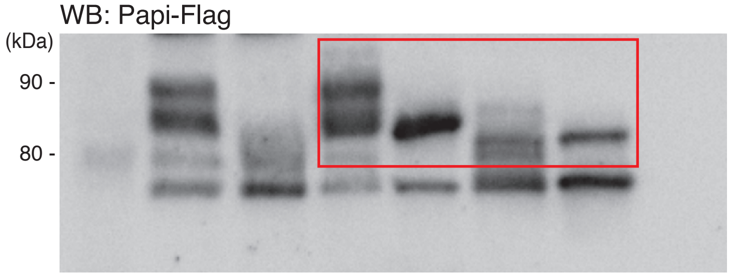

Fig. 2c

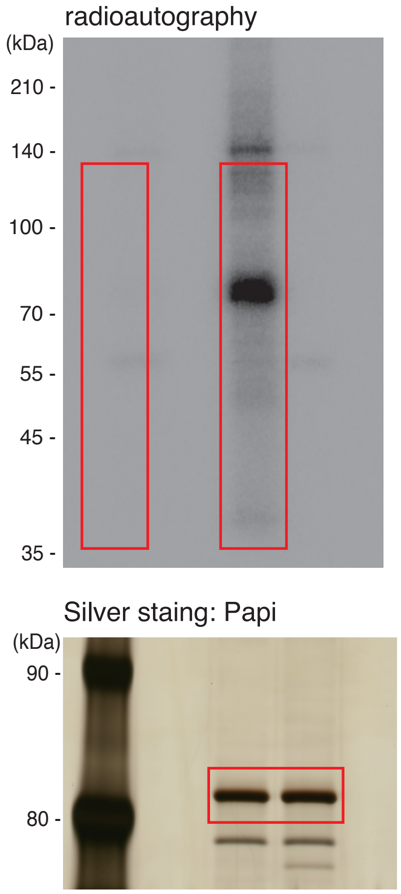

Fig. 2d

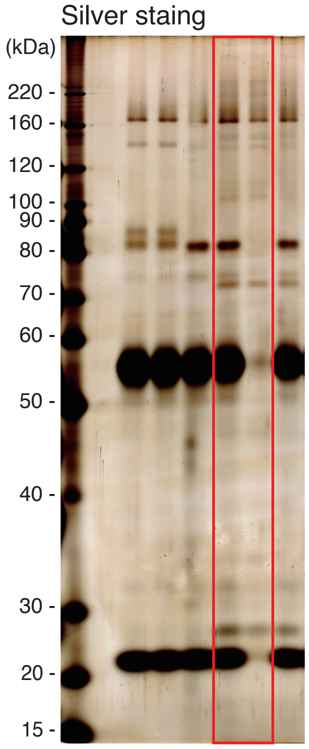

Fig. 2f

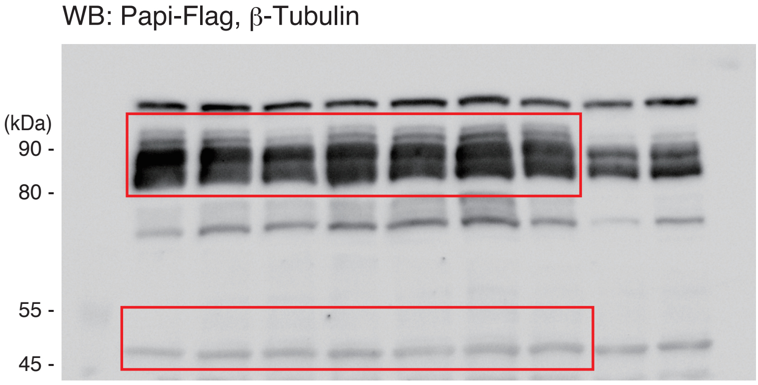

Fig. 2g

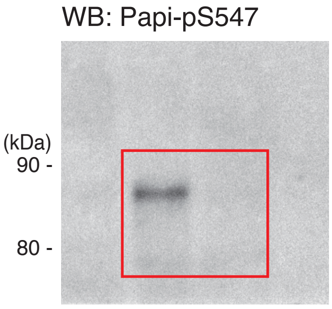

Fig. 2h

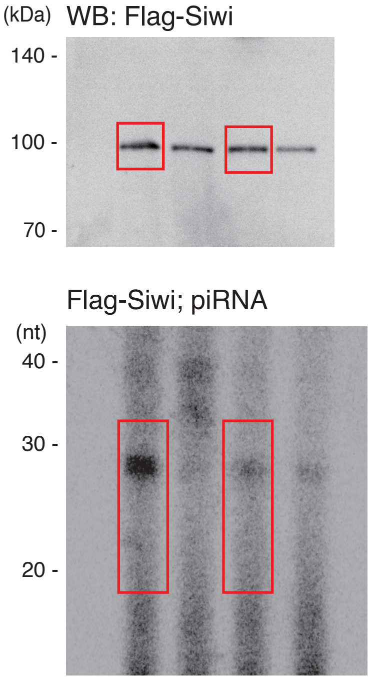

Original source data of blotting images of Figure.3

Fig. 3a

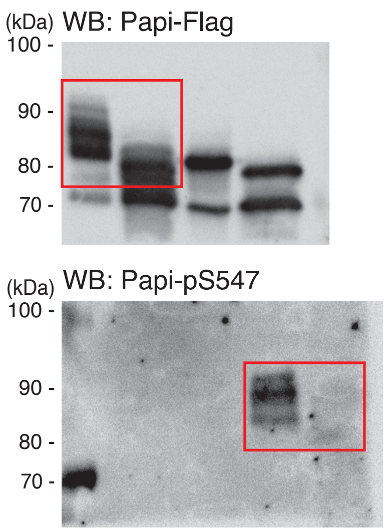

Fig. 3b

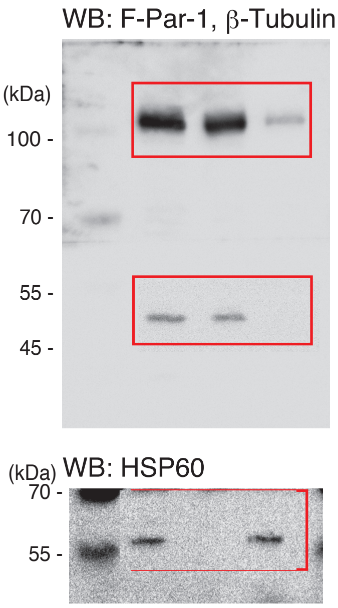

Fig. 3c

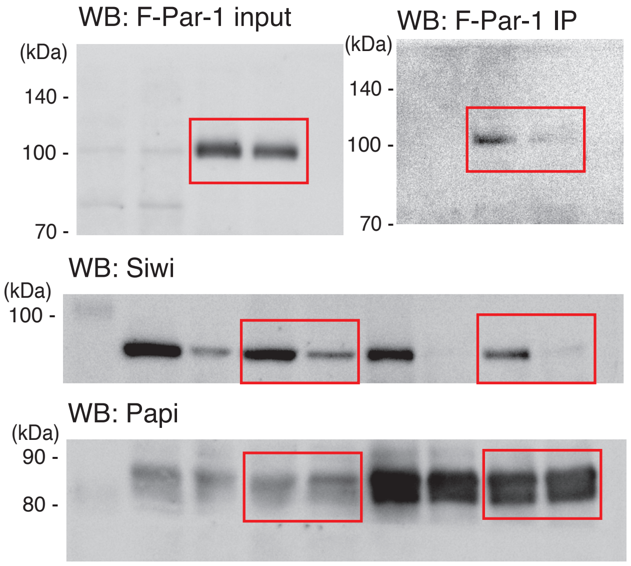

Fig. 3d

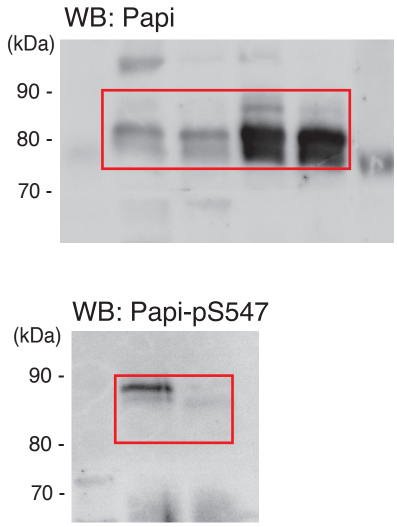

Fig. 3e

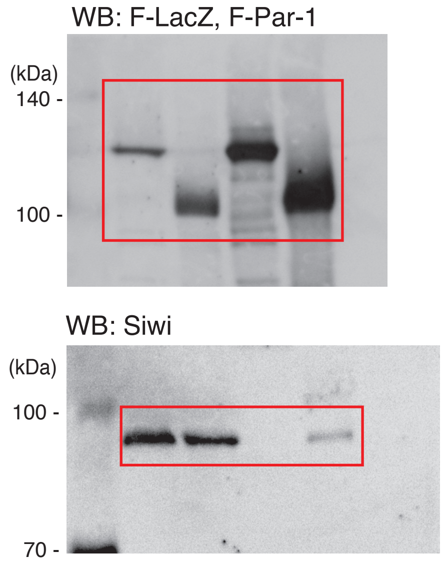

Fig. 3f

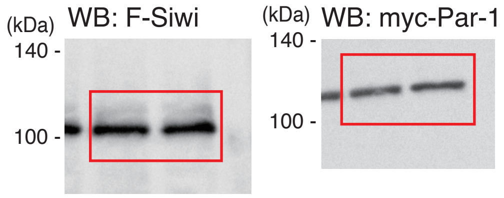

Fig. 3g

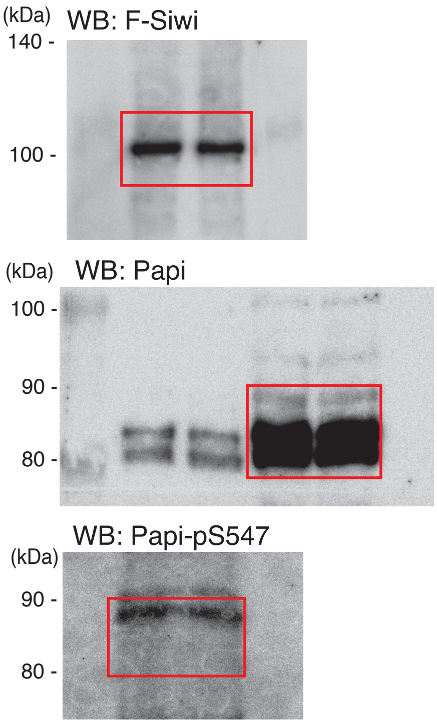

Fig. 3i

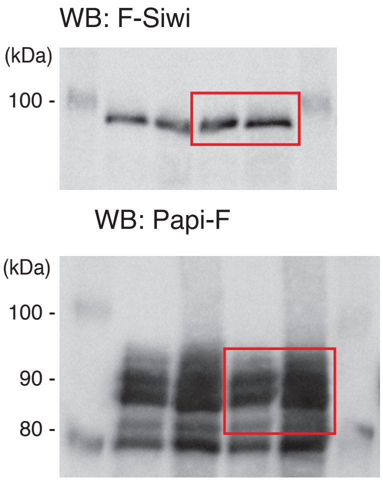

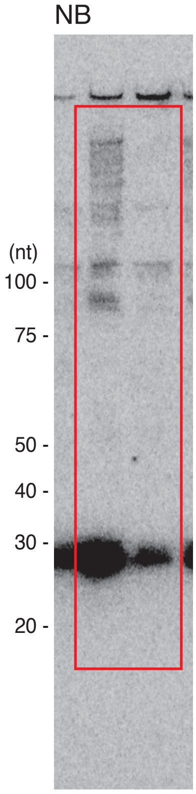

Original source data of blotting images of Figure.4

Fig. 4a

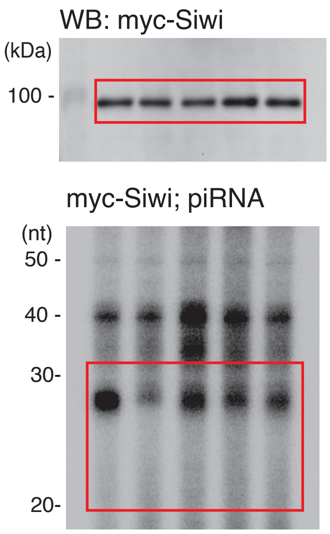

Fig. 4b

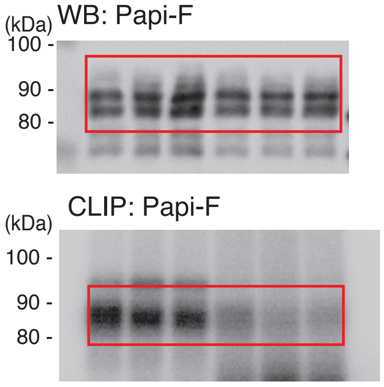

Fig. 4c

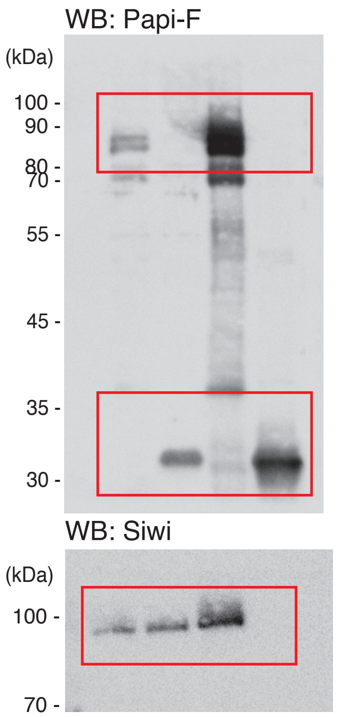

Fig. 4d

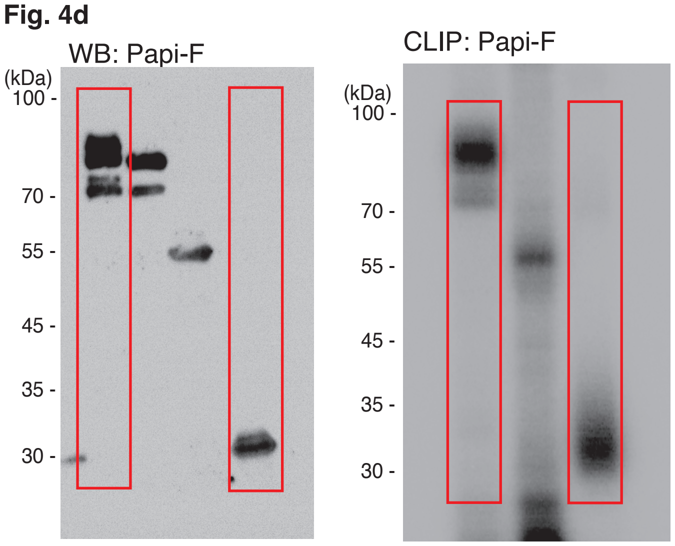

Fig. 4f

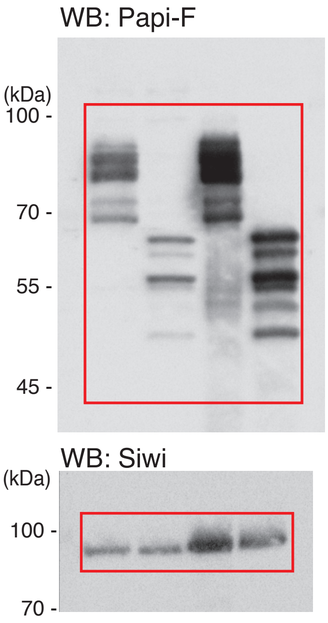

Fig. 4g

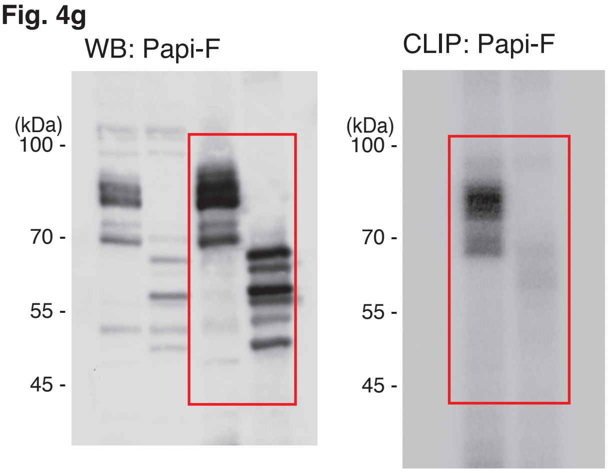

Original source data of blotting images of Supplementary Figures

Supplementary Fig.1d

WB: Siwi, Papi-F

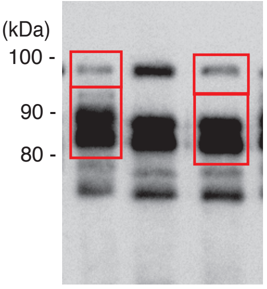

Supplementary Fig.1e

WB: myc-Siwi

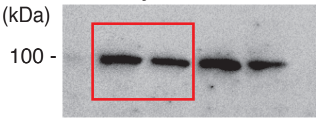

WB: Papi

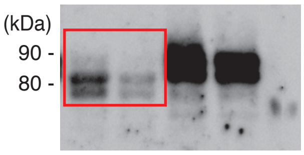

Supplementary Fig.2a

WB: β-Tubulin

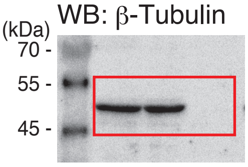

WB: HSP60

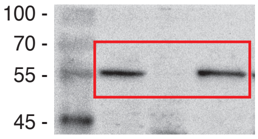

Supplementary Fig.2d

WB: Papi

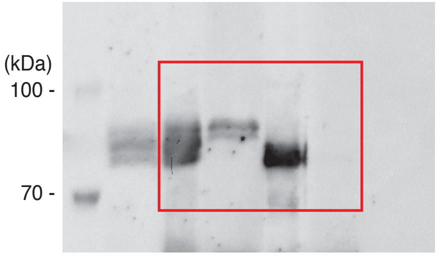

Supplementary Fig.2e

WB: Papi

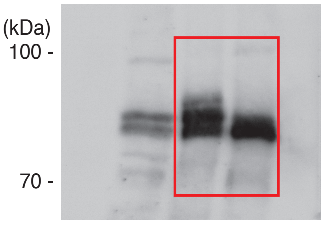

WB: Papi-pS547

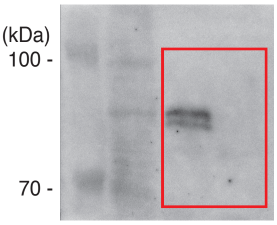

Supplementary Fig.2f

WB: Papi-pS547

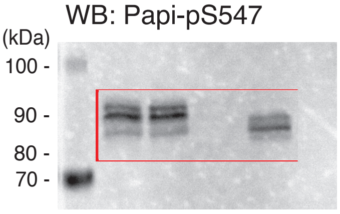

Supplementary Fig.2g

WB: Papi-pS547

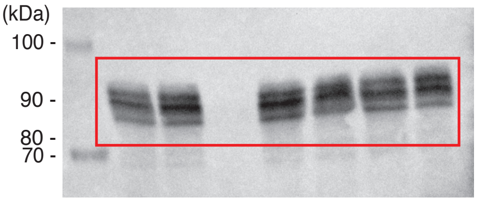

Supplementary Fig.4a

WB: myc-Siwi, Papi

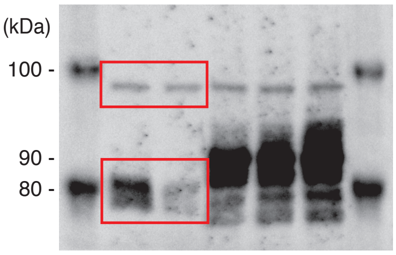

Supplementary Fig.4b

WB: Siwi

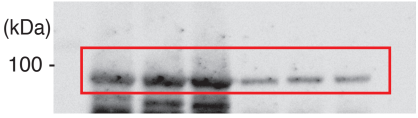

Supplementary Fig.4c

WB: Papi-F

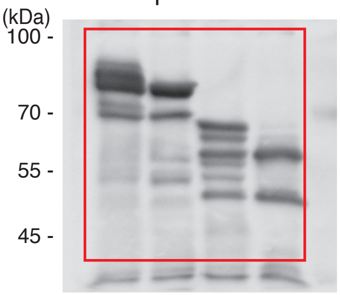

Supplementary Fig.4d

WB: Papi-F

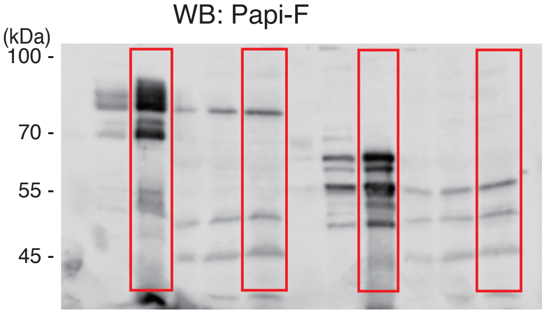

Supplement: Supplementary file 4 — Source Data [file 41467_2022_29193_MOESM4_ESM.zip › Source data_blot_Yamada et al.pdf]
